# CCL18-NIR1 promotes oral cancer cell growth and metastasis by activating the JAK2/STAT3 signaling pathway

**DOI:** 10.1186/s12885-020-07073-z

**Published:** 2020-07-08

**Authors:** Xiao Jiang, Zhijie Huang, Xiang Sun, Xianghuai Zheng, Jingpeng Liu, Jun Shen, Bo Jia, Haiyun Luo, Zhaoyi Mai, Guodong Chen, Jianjiang Zhao

**Affiliations:** 1 Stomatology Center, Shunde Hospital, Southern Medical University (The First People’s Hospital of Shunde), Foshan, Guangdong China; 2 Stomatological Hospital, Southern Medical University, Guangzhou, Guangdong China

**Keywords:** Oral squamous cell carcinoma, CCL18, NIR1, JAK2/STAT3

## Abstract

**Background:**

Chemokine (C-C motif) ligand 18 (CCL18) affects the malignant progression of varying cancers by activating chemokine receptors. Our previous work has shown that CCL18 promotes hyperplasia and invasiveness of oral cancer cells; however, the cognate receptors of CCL18 involved in the pathogenesis of oral squamous cell carcinoma (OSCC) have not yet been identified. This study aimed to investigate the molecular mechanisms which underlie promotive effects of CCL18 on OSCC progression by binding to functional receptors.

**Methods:**

The expression of CCL18 receptor-NIR1 in OSCC was determined by conducting western blot, immunofluorescence, and immunocytochemistry assays. Chi square test was applied to analyze the relationship between expression levels of NIR1 and clinicopathological variables. Recombinant CCL18 (rCCL18), receptor siRNA and JAK specific inhibitor (AG490) were used in experiments investigating the effects of the CCL18-NIR1 axis on growth of cancer cells (i.e., proliferation, and metastasis), epithelial-mesenchymal transition (EMT) and the activation of the JAK2/STAT3 signaling pathway.

**Results:**

NIR1 as functional receptor of CCL18 in OSCC, was found to be significantly upregulated in OSCC and positively related to the TNM stage of OSCC patients. rCCL18 induced the phenotypical alterations in oral cancer cells including cell growth, metastasis and EMT. The JAK2/STAT3 signaling pathway was confirmed to be a downstream pathway mediating the effects of CCL18 in OSCC. AG490 and knockdown of NIR1 could block the effects of rCCL18-induced OSCC.

**Conclusion:**

CCL18 can promote the progression of OSCC by binding NIR1, and the CCL18-NIR1 axis can activate JAK2/STAT3 signaling pathway. The identification of the mechanisms underlying CCL18-mediated promotion of OSCC progression could highlight potential therapeutic targets for treating oral cancer.

## Background

Oral squamous cell carcinoma (OSCC) is the most common type of oral cancer and is well-understood as characterized by a high risk of local invasion and cervical lymph node metastasis. As a matter of concern, the 5-year survival rate of patients with oral cancer remains less than 50% and has not seen significantly improvement in recent decades despite advances in treatment approaches [1, 2]. Chemokines play important mechanistic roles in tumor development and have been shown to promote metastasis in OSCC by facilitating the proliferation, survival, and migration of cancer cells, as well as by alteration of the tumor microenvironment [3–5]. Our previous work have shown that the dysregulation of chemokine (C-C motif) ligand 18 (CCL18) is involved in the development of OSCC by promoting the growth and invasion of cancer cells [6]. However, the mechanisms underlying of CCL18-mediated promotion of OSCC remain unclear.

Chemokines have been found to elicit their effects mainly by activating specific transmembrane receptors which belong to the large family of G protein-coupled receptors (GPCRs) [7]. The chemokines-receptor signaling axis has been therefore considered as a hallmark of cancer and the basis for potential therapeutic strategy development [8]. NIR1 (PYK2 N-terminal domain interacting receptor 1, also named phosphatidylinositol transfer protein 3, PITPNM3) has been verified as the most common functional receptor of CCL18. NIR1 can bind to CCL18, which further stimulates calcium signaling, and finally elicits a cancer-promoting function in various malignancies (e.g., breast cancer, non-small cell lung cancer and ovarian cancer) [9–11]. However, the role of the CCL18/NIR1 axis in OSCC is unclear. In addition, CCR8 and CCR6 have also been reported as CCL18 receptors in several immune disease and tumors. The CCL18-CCR8 axis enhances the migration, invasion and EMT in bladder cancer [12]. CCL18 binding to CCR6 enhances pulmonary fibrosis by human lung fibroblasts [13]. Whether CCR8 and CCR6 play significantly impact mechanistic roles in OSCC is yet not understood.

As NIR1 has been identified as a specific receptor of CCL18, the putative role of the CCL18-NIR1 axis in regulating OSCC emerges as a significant research question. In addition, investigating the downstream signaling pathways involved also assumes importance. In this regard, the JAK2/STAT3 (Janus kinase 2/signal transducers and activators of transcription 3) signaling pathway is an oncogenic pathway implicated in many solid cancers including OSCC [14]. In particular, it has been shown to be activated by several chemokine-siganling axes, has been shown to be activated by several chemokine-signaling axes, for instance, CXCL12-CXCR4 axis [15], CXCL8-CXCR1/CXCR2 axis [16], and CXCL9-CXCR3 axis [17]. The interactions between JAK2/STAT3 and chemokine-receptor axes thus appear of significant interest in context of molecular mechanisms of OSCC. Therefore, in the present study we investigated the putative role of the CCL18-NIR1 signaling axis in OSCC. Furthermore, we aimed to examine if it is coupled with the JAK2/STAT3 pathway, and, if an interaction of these pathways contributes mechanistically to metastasis of OSCC.

## Methods

### Patients and samples

Twenty-five patients with OSCC underwent surgical resection at the Department of Craniofacial Surgical Resection, Stomatological Hospital, Southern Medical University, Guangzhou, China. Primary OSCC tissues (*n* = 25) and some adjacent normal tissues (*n* = 10) were obtained postoperatively. All patients provided written informed consent prior to enrolment in the study. The study protocol was approved by the Ethics Committee of Stomatological Hospital, Southern Medical University. Another 18 OSCC tissue samples were acquired from tissue chips with detailed clinical information and were purchased from WoZhe Biotechnology Company Ltd. (Guangzhou, China).

### Cell lines and reagents

The HSC6 cell line was purchased from CinoAsia Co., Ltd. (Shanghai, China). CAL27, SCC9 and HOK cell lines were purchased from TongPai Biotechnology Co., Ltd. (Shanghai, China). OSCC cells were maintained in Dulbecco’s modified Eagle’s medium (DMEM, Gibco, Grand Island, NY, USA) supplemented with 10% foetal bovine serum (FBS, Gibco, USA) and 1% penicillin-streptomycin, and HOK cells were cultured in KSFM (Gibco, USA). Cells were incubated in a humidified atmosphere of 5% CO2 at 37 °C. Recombinant human CCL18 (rCCL18) was obtained from Peprotech (Princeton, NJ, USA). The JAK2/STAT3 signaling pathway specific inhibitor AG490 was purchased from Selleck Chemicals (Houston, TX, USA).

### Immunohistochemistry

The OSCC tissues and adjacent normal tissues each were analyzed using immunohistochemistry (IHC). In brief, tissues were dewaxed in xylene and rehydrated using a graded alcohol series. After antigen retrieval with Tris-EDTA, the slides were blocked with 5% serum. Primary antibodies against CCL18 (1:100, Santa Cruz Biotechnology Inc., USA), NIR1 (1:100, Novus, Littleton, CO, USA), CCR6 (1:100, Novus, Littleton, CO, USA) and CCR8 (1:100, Abcam, UK) were incubated overnight at 4 °C. Then, the sections were covered with secondary antibody and incubated at room temperature for 30 min. Next, the tissue sections were visualized with DAB (Gene, Shanghai, China). The staining results were evaluated using a visual grading system based on the average optical density scored using the following criteria: the percent score of positive cells: 0 (< 5%); 1 (5–25%); 2 (26–50%); 3 (51–75%); 4 (76–100%); the staining intensity: 0 (negative), 1 (weak), 2 (moderate), 3 (strong). Positive grade = percentage score × staining intensity score. Specifically, 0–1 was considered as (−), 2–8 as (+), 9–12 as (++).

### Immunofluorescence

Cells were seeded in glass bottom cell culture dishes for 24 h. Thereater, the cells were rinsed with PBS, fixed with 4% paraformaldehyde solution for 30 min, permeabilized with 0.3% Triton X-100 for 15 min, and blocked with 5% bovine serum albumin (BSA) for 1 h. Subsequently, the cells were incubated overnight at 4 °C with the following primary antibodies: NIR1 (1:200, Novus, USA), CCR6 (1:200, Novus, USA), and CCR8 (1:100, Abcam, UK). The next day, the samples were incubated with secondary antibody (1:500, Abcam, UK) in the dark for 1 h and counterstained with DAPI (Invitrogen, USA) for 5 min. The results were photographed using an automated upright microscope system (Leica, DM4000B Leica Microsystems, Wetzlar, Germany).

### Transfection of NIR1 siRNA

For transfection, HSC6 cells and CAL27 cells were seeded in 6-well plates at 2 × 10^5^/well. siRNA against NIR1 (siNIR1) was transferred into cells with Lipofectamine 2000 (Invitrogen, USA), used according to the manufacturer’s instructions. A negative siRNA (siNC) sequence was used as a control. Silencing efficiency was verified by qRT-PCR and Western blot assays after 48 h of transfection. The following three interfering sequences for NIR1 were synthesized (GenePharma, Jiangsu, China):

siNIR1–1: sense 5′-CCAUCUGCUCUGAGGCUUUTT-3′ antisense 5′-AAAGCCUCAGAGCAGAUGGTT-3′.

siNIR1–2: sense 5′-CACGCCCAAAGAAGAACAATT-3′ antisense 5′-UUGUUCUUCUUUGGGCGUGTT-3′.

siNIR1–3: sense 5′-GUGGUCGCAUCACAUACAATT-3′ antisense 5′-UUGUAUGUGAUGCGACCACTT-3′.

Negative control: sense 5′-UUCUCCGAACGUGUCACGUTT-3′ antisense 5′-ACGUGACACGUUCGGAGAATT-3′.

### Western blot analysis

Cells and tissues were lysed in cell lysis buffer with phosphatase inhibitor, protease inhibitor and PMSF (KeyGEN BioTECH, Jiangsu, China). Total protein levels were measured using a BCA protein assay kit (Cwbiotech, Jiangsu, China). Twenty micrograms of protein was separated by 10% SDS-PAGE and transferred onto a PVDF membrane (Merck KGaA, Darmstadt, Germany). The PVDF membrane was blocked with 5% BSA (Pierce, Rockford, IL, USA) for 1 h and then incubated with the following primary antibodies at 4 °C overnight: NIR1 (1:2000, Novus, USA), CCR6 (1:250, Novus, USA), CCR8 (1:2000, Abcam, UK), GAPDH (1:1000, Abcam, UK), E-cadherin (1:1000, CST, Danvers, MA, USA), N-cadherin (1:1000, CST, USA), ZEB2 (1:1000, Merck KGaA, Germany), JAK2 (1:1000, CST, USA), P-JAK2 (Tyr1007/1008) (1:1000, CST, USA), STAT3 (1:1000, Sant Cruze Biotechnology Inc., USA), P-STAT3 (Tyr705) (1:1000, CST, USA), and β-actin (1:1000, Abcam, UK). Thereafter, the PVDF membrane was incubated with secondary antibody (1:2000, Abcam, UK). Protein bands were detected by ultrasensitive chemiluminescence imaging, and Image Lab software was used to analyse the density of each band.

### qRT-PCR

Cells were collected, and total RNA was extracted using TRIzol reagent (Invitrogen, USA). Complementary DNA (cDNA) was synthesized using a FastKing gDNA Dispelling RT SuperMix (TIANGEN, Beijing, China). qPCR was performed using the Talent qPCR PreMix (TIANGEN, China) on a CFX96TM Connect Real-Time System (C1000 TouchTM Thermal Cycler, BIO-RAD, Hercules, CA, USA). The thermocycling conditions were as follows: 3 min at 95 °C, followed by 40 cycles of 5 s at 95 °C and 15 s at 60 °C. The relative levels of mRNA expression were normalized to GAPDH levels as the reference gene, using the 2^-∆∆Cq^ method.

The primers sequences used were as follows: NIR1: (Forward: GATGCCAGAGGAGAAGGGAC; Reverse: TCGCTGTCTTCGTGGATCTC), GAPDH: (Forward: CTCCTCCTGTTCGACAGTCAGC; Reverse: CCCAATACGACCAAATCCGTT).

### CCK-8 assay

Cells were pretreated with siRNA-NIR1for 48 h or AG490 for 24 h, and 5000 cells were then added to 96-well plates and treated with 20 ng/ml rCCL18. At 24 h, 48 h and 72 h, CCK-8 reagent (Sigma-Aldrich, Louis, MO, USA) was added, and the absorbance values of each well at 450 nm were read using a microplate reader (Thermo Fisher Scientific. Waltham, USA).

### Clone formation assay

Forty-eight hours after siRNA-NIR1 transfection, cells were plated in 6-well plates at 1000 cells per well and exogenously stimulated with 20 ng/ml rCCL18 (3% FBS). The number of cell clones was counted using crystal violet staining 14 days later.

### Transwell assays

Cell migration and invasion were detected using transwell assays (Corning, New York, NY, USA). The upper chamber was precoated with 50 μl 20% Matrigel (Gibco, USA) for the invasion assay. Cells were transfected with siRNA for 48 h or treated with AG490 for 24 h. Treated cells were suspended in serum-free medium with or without 20 ng/ml rCCL18. The prepared cells were seeded in the upper insert, and the lower chamber was filled with DMEM containing 15% FBS. Then, the transwell plates were incubated at 37 °C with 5% CO_2_ for 24 h. Cells that did not invade through the pores were gently removed with cotton tips. The upper chamber was fixed with 4% formaldehyde for 15 min and stained with a 0.4% crystal violet solution for 15 min. Five randomly selected fields of view at × 50 magnification were photographed under a light microscope (Carl Zeiss AG, Oberkochenm, Germany) and analyzed.

### Statistical analysis

Statistical analysis was performed using GraphPad Prism 7.00 software (GraphPad Software, Inc., La Jolla, CA, USA) and SPSS version 20 (IBM Corporation, Armonk, NY, USA). The data are presented as means±SEM based on three replicates per group. Chi square tests was used to analyze the association of NIR1 with the clinical variables of OSCC patients. Student’s t test and one-way ANOVA were used to compare the mean differences between different sample group. *P* < 0.05 was considered statistically significant.

## Results

### NIR1 expression in OSCC and its clinical significance

**Fig. 1:**
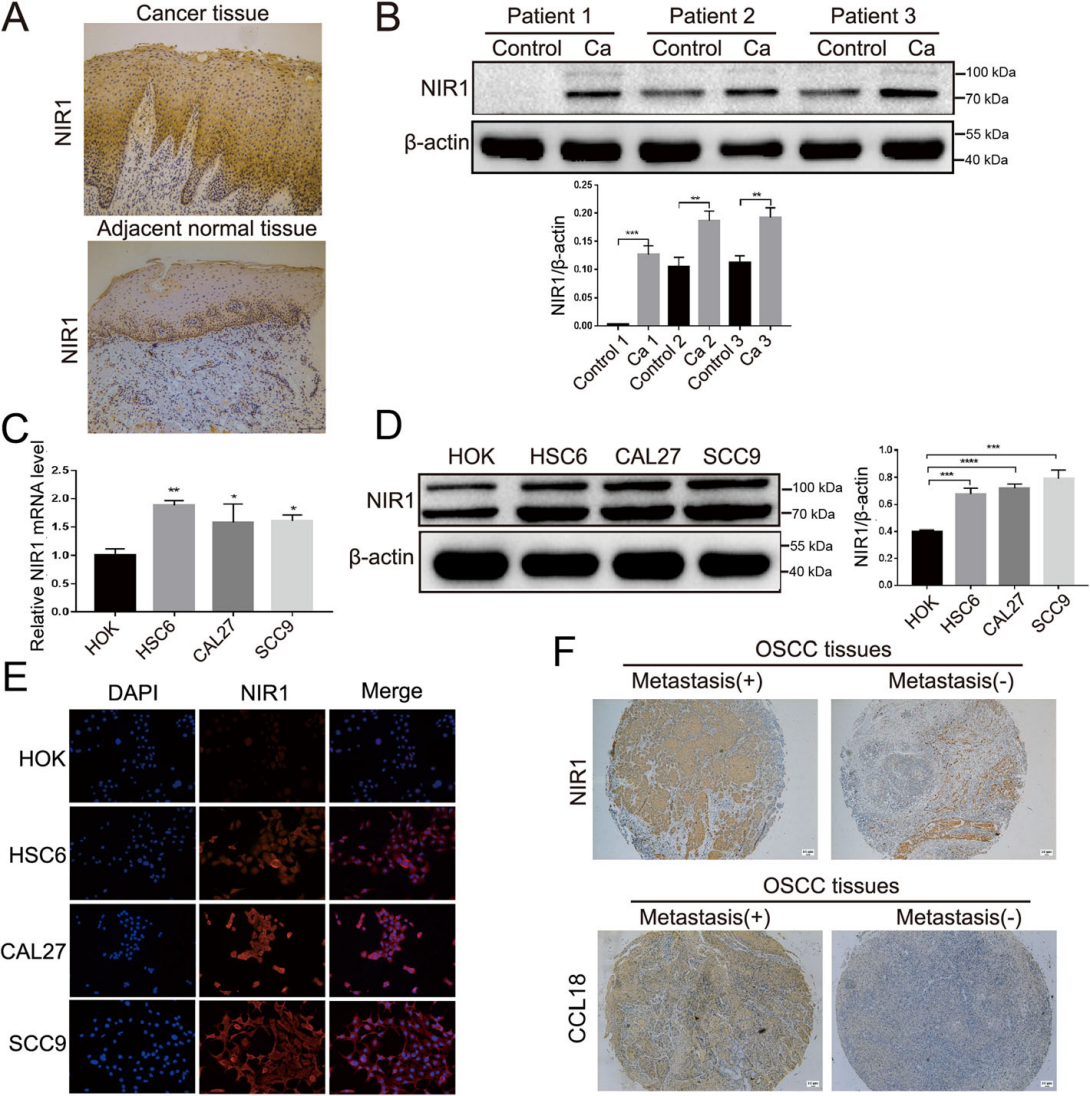
NIR1, which is a potential receptor of CCL18, was shown to be highly expressed in OSCC. **a** Representative images of NIR1 staining in OSCC tissues and adjacent normal tissues (magnification 100×). **b** Western blotting results showed that the protein level of NIR1 is up-regulated in oral cancer tissues (**P < 0.01, ***P < 0.001 vs. adjacent normal tissues). **c** and **d** qRT-PCR assay and western blotting showed that NIR1 was overexpressed in OSCC cell lines (i.e., HSC6, CAL27, SCC9) compared with HOK cells (*P < 0.05, **P < 0.01, ***P < 0.001, ****P < 0.0001 vs. HOK). The data represent mean ± SEM of three independent experiments. **e** Immunofluorescence staining of NIR1 (red) and nuclei (blue) in oral cancer cells (i.e., HSC-6, CAL27 and SCC9) and HOK cells (magnification 200×). **f** IHC images showed that NIR1 and CCL18 more expressed in metastatic cases of OSCC than in non-metastatic cases (magnification 50×). The full-length blots are presented in Supplementary Fig. S1

NIR1 is the most common receptor of CCL18, and their potent combination has been verified in breast cancer [18]. To investigate the role of NIR1 in OSCC, immunohistochemistry (IHC) and western blot assay were performed to determine the NIR1 expression pattern in 10 pairs of OSCC tissues and adjacent normal tissues. The results of IHC revealed that positive staining for NIR1 was primarily localized in the cellular membrane and cytoplasm of oral cancer cells (Fig. 1a). In addition, western blot results showed that the expression of NIR1 was significantly higher in cancer tissues than that in adjacent normal tissues (Fig. 1b, Fig. S1). Furthermore, we examined the expression levels of NIR1 in 3 OSCC cell lines (HSC6, CAL27 and SCC9) and in normal human oral epithelial keratinocytes (HOK). qRT-PCR, western blot, and immunofluorescence (IF) assays verified that NIR1 was highly expressed in all OSCC cells as compared to HOK cells (Fig. 1c-e, Fig. S1).

To further evaluate the clinical significance of NIR1, 43 OSCC tissues were analyzed using NIR1 antibodies and CCL18 antibodies for IHC. All OSCC tissues displayed positive NIR1 expression. Highly NIR1-positive tissues were significantly associated with the clinical TNM stage (*P* = 0.042, Table 1; Fig. 1f). Moreover, the expression of NIR1 in OSCC tissues was significantly correlated with those of CCL18 (*r* = 0.440, *P* = 0.003, Table 1). However, there was no significant relationship between NIR1 expression levels and other clinical features, such as age, sex or histological grade of OSCC patients.

**Table 1:**
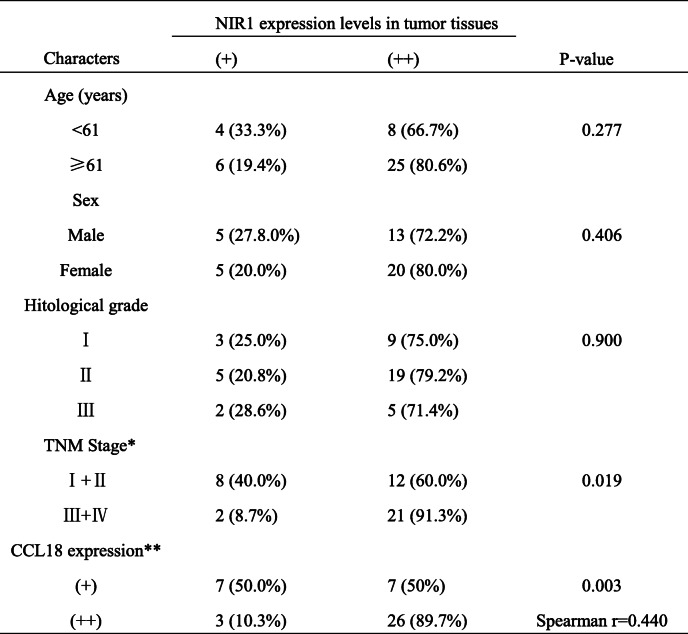
Association of NIR1 expression with the clinicopathological characteristics in OSCC

*Based on the American Joint Committee on Cancer (AJCC, 8th Edition)

**Fig. 2:**
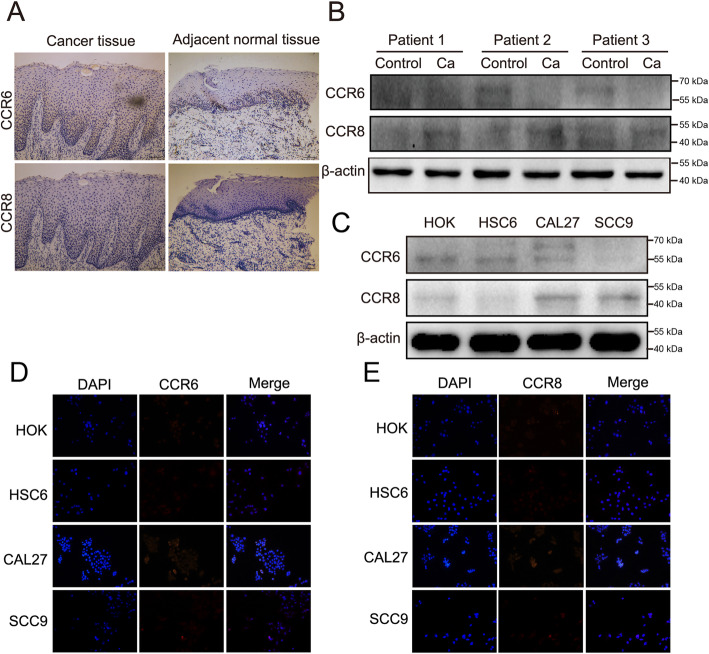
Negative expression of CCR6 and CCR8 in OSCC. **a** Representative images of CCR6 and CCR8 staining in OSCC tissues and adjacent healthy tissues (magnification 100×). **b** and **c** Results of western blot showed that CCR6 and CCR8 were rarely expressed in OSCC tissues, adjacent healthy tissues, OSCC cells, and HOK cells. The full-length blots are presented in Supplementary Fig. S2. **d** and **e** Immunofluorescence staining of CCR6 and CCR8 (red) and nuclei (blue) in oral cancer cells (HSC-6, CAL27 and SCC9) and HOK cells (magnification 200×)

CCR6 and CCR8 have also been reported as CCL18 receptors involved in the development of various malignancies. In this study, we also determined the expression levels of CCR6 and CCR8 in oral cancer. Interestingly, low protein expression levels of CCR6 and CCR8 were found in cancer tissues and adjacent tissues (Fig. 2a, b, Fig. S2). In agreement, in-vitro results form cells models also verified that CCR6 and CCR8 were both rarely expressed in both OSCC cells and HOK cells (Fig. 2c-e, Fig. S2).

### Effective interference sequences of NIR1 in OSCC cells

**Fig. 3:**
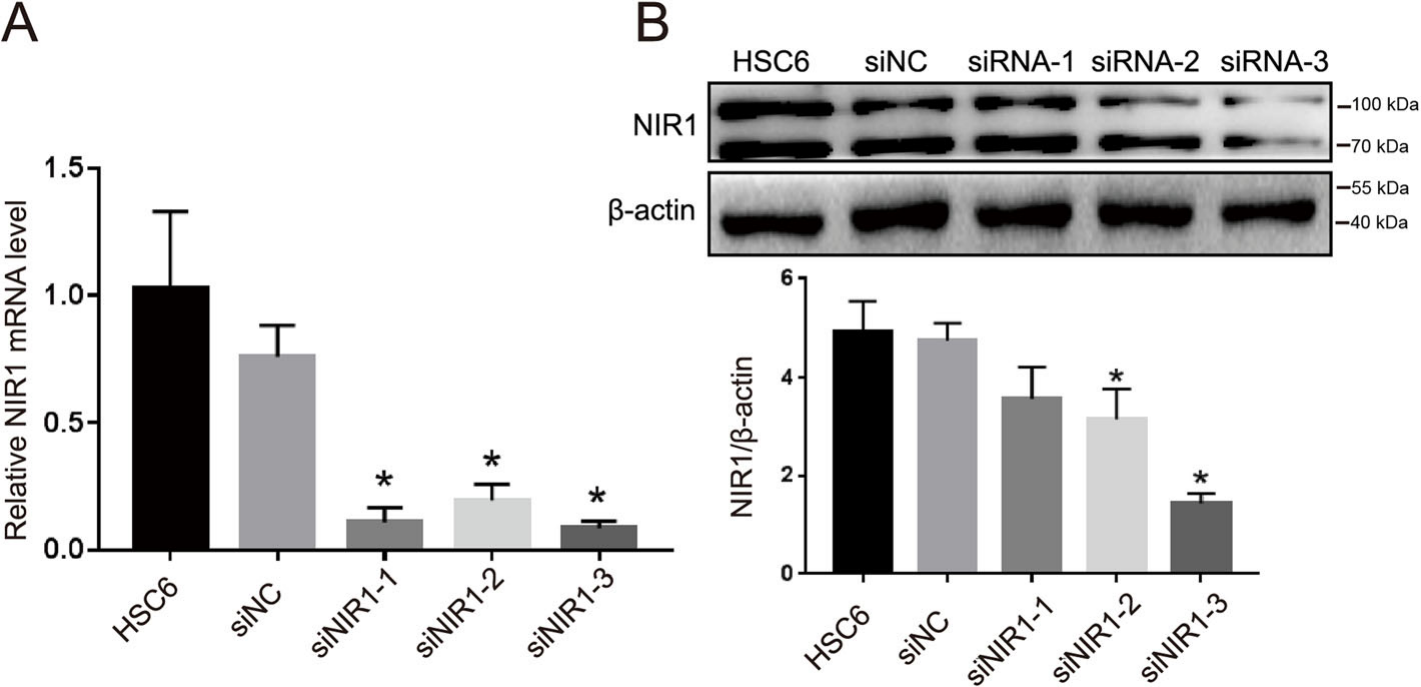
Effective siRNA segments of NIR1 were screened in HSC6 cells. qRT-PCR and western blotting measured the mRNA expression **a** and NIR1 protein expression **b** in HSC6 cells, which were transfected with siNIR1–1, siNIR1–2, siNIR1–3, respectively (*P < 0.05 vs. siNC control). The full-length blots are presented in Supplementary Fig. S3

We next sought to confirm that CCL18 regulates the progression of OSCC through NIR1. Three different siNIR1 segments were designed to screen effective interference sequences. Our results showed that the mRNA levels of NIR1 was significantly decreased by siNIR1–1, siNIR1–2, and siNIR1–3 (Fig. 3a). Western blot assays showed that siNIR1–2 and siNIR1–3 could decrease the protein levels of NIR1 (Fig. 3b, Fig. S3). However, there were no significant changes in mRNA or protein levels in untreated cells or cells transfected with negative siRNA sequences. Overall, these data suggested that siNIR1–3 was an effective sequence suitable for subsequent experiments.

### NIR1 is required for OSCC cell proliferation via CCL18

**Fig. 4:**
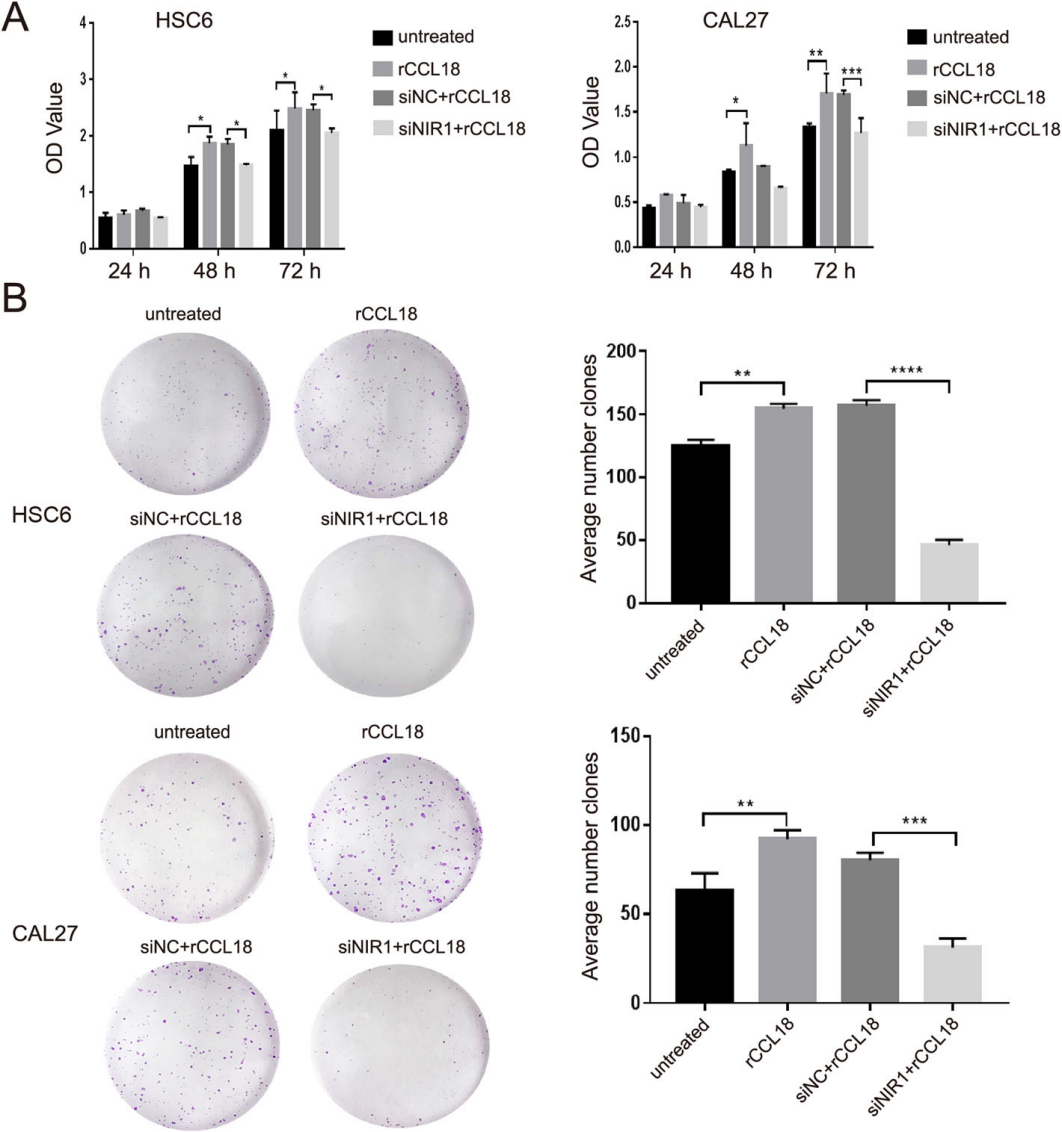
CCL18-NIR1 axis affected the proliferation of OSCC cells. Cells were treated with rCCL18, siNC+rCCL18, and siNIR1 + rCCL18, respectively. The untreated cells and siNC+rCCL18 cells were used as control. **a** CCK8 assay analyzed the proliferation of cells in different treatment groups at 24 h, 48 h, and 72 h. **b** HSC6 cells and CAL27 cells were treated as above. Cells were under continuous stimulation of 20 ng/ml rCCL18 for 14 days and also were stained with 0.4% crystal violet. (*P < 0.05, **P < 0.01, ***P < 0.001, ****P < 0.0001 vs. control)

To investigate the function of the CCL18-NIR1 axis in OSCC, HSC6 and CAL27 cells were transfected with siNIR1 and then stimulated with 20 ng/ml rCCL18. The proliferation of each group of cells was determined by CCK8 and clone formation assays. CCK8 assays showed that the proliferation of OSCC cells increased upon rCCL18 stimulation for 48 h and 72 h. However, upon knocking down NIR1, the proliferative effect of rCCL18 on OSCC cells was reduced (Fig. 4a). As shown in Fig. 4b, OSCC cells cultured with rCCL18 showed strong clone formation ability for 14 days. Compared to the control conditions, transfection with siNIR1 of cells cultured with rCCL18 resulted in a significant decrease in colony numbers.

### NIR1 is required for cell mobility and EMT in OSCC cells via CCL18

**Fig. 5:**
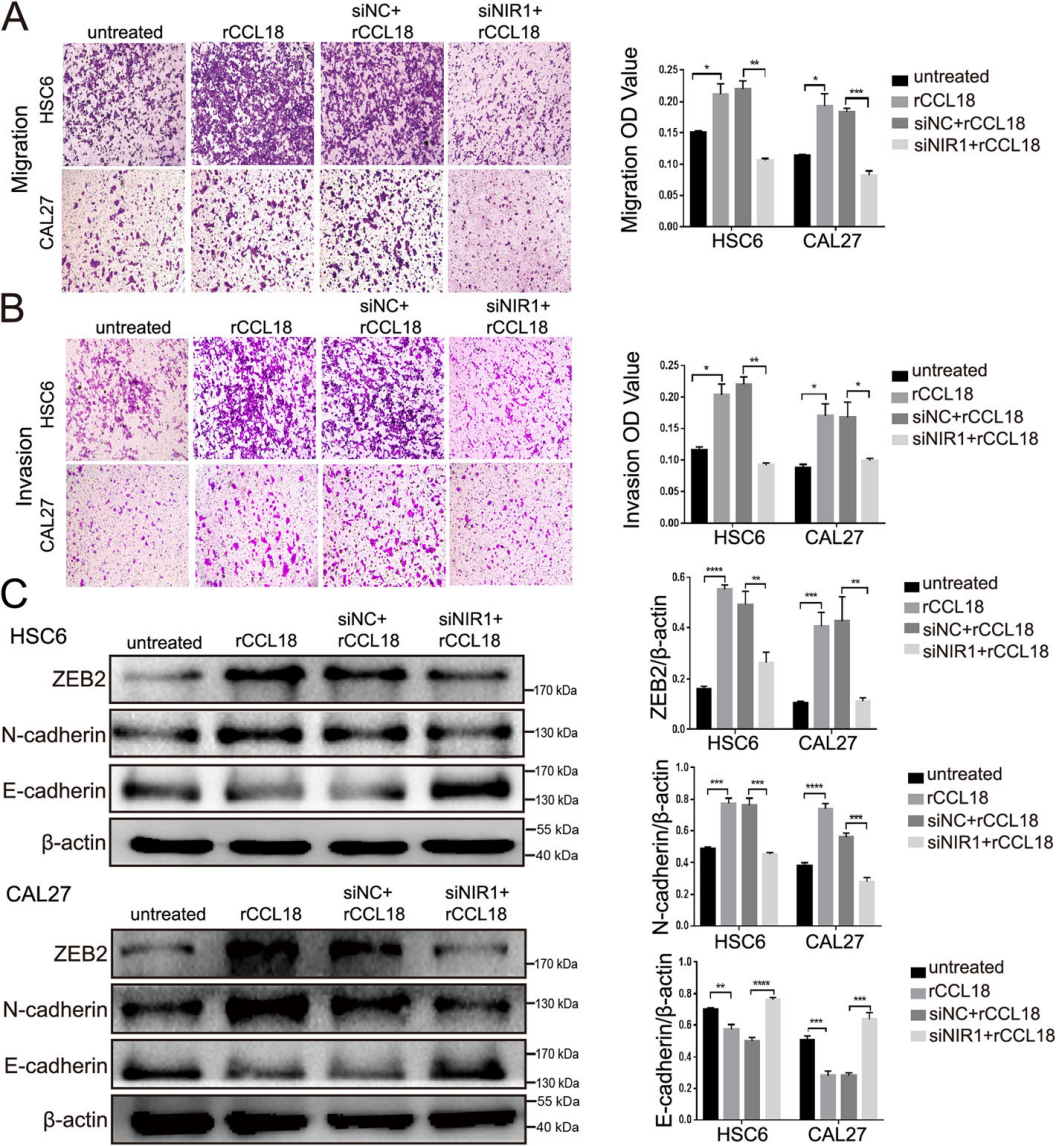
CCL18-NIR1 axis promoted the locomotion and EMT of OSCC cells. HSC6 cells and CAL27 cells were treated with rCCL18, siNC+rCCL18 and siNIR1 + rCCL18 respectively. The untreated cells and siNC+rCCL18 cells were used as control. **a** and **b** The effects of CCL18-NIR1 axis on the migration and invasion of OSCC cells. Oral cancer cells at the bottom of the transwell membrane were stained with 0.4% crystal violet. Five fields per hole were randomly selected under the microscope at 50×. **c** The protein expression of E-cadherin, N-cadherin and ZEB2 in HSC6 and CAL27 cells which subjected to the different treatment stated above were tested by western blotting, β-actin was used as the internal control. (*P < 0.05, **P < 0.01, ***P < 0.001, ****P < 0.0001 vs. control). The full-length blots are presented in Supplementary Fig. S4

As NIR1 was associated with the tumor stage of OSCC patients, we characterized the effects of the CCL18-NIR1 axis on the migration and invasion of OSCC cells, using a transwell assay. HSC6 and CAL27 cells were treated with rCCL18, siNIR1 + rCCL18, while untreated cells and siNC+rCCL18 treatment served as controls. The results depicted in Fig. 5a showed that rCCL18 could promote OSCC cell migration through the transwell membrane. In the presence of siNIR1, the number of cells on the submembrane surface decreased despite rCCL18 stimulation. Similar results were observed in the cell invasion assay (Fig. 5b).

EMT plays a critical role in oral cancer metastasis by enhancing migration and invasion. Therefore, we examined the expression of EMT markers, E-cadherin, N-cadherin and ZEB2 in HSC6 and CAL27 cells subjected to the different treatments stated above. Compared with that in the untreated group, the expression of E-cadherin decreased, and the expression of N-cadherin and ZEB2 increased in the rCCL18 group. Knocking down NIR1 reversed the decreases in E-cadherin levels and the increases in N-cadherin and ZEB2 levels caused by rCCL18 (Fig. 5c, Fig. S4). These data demonstrated that CCL18 enhanced EMT in OSCC cells by binding to NIR1 and thereby promoting OSCC cell invasion and migration.

### CCL18-NIR1 axis activates the JAK2/STAT3 signaling pathway in OSCC cells

**Fig. 6:**
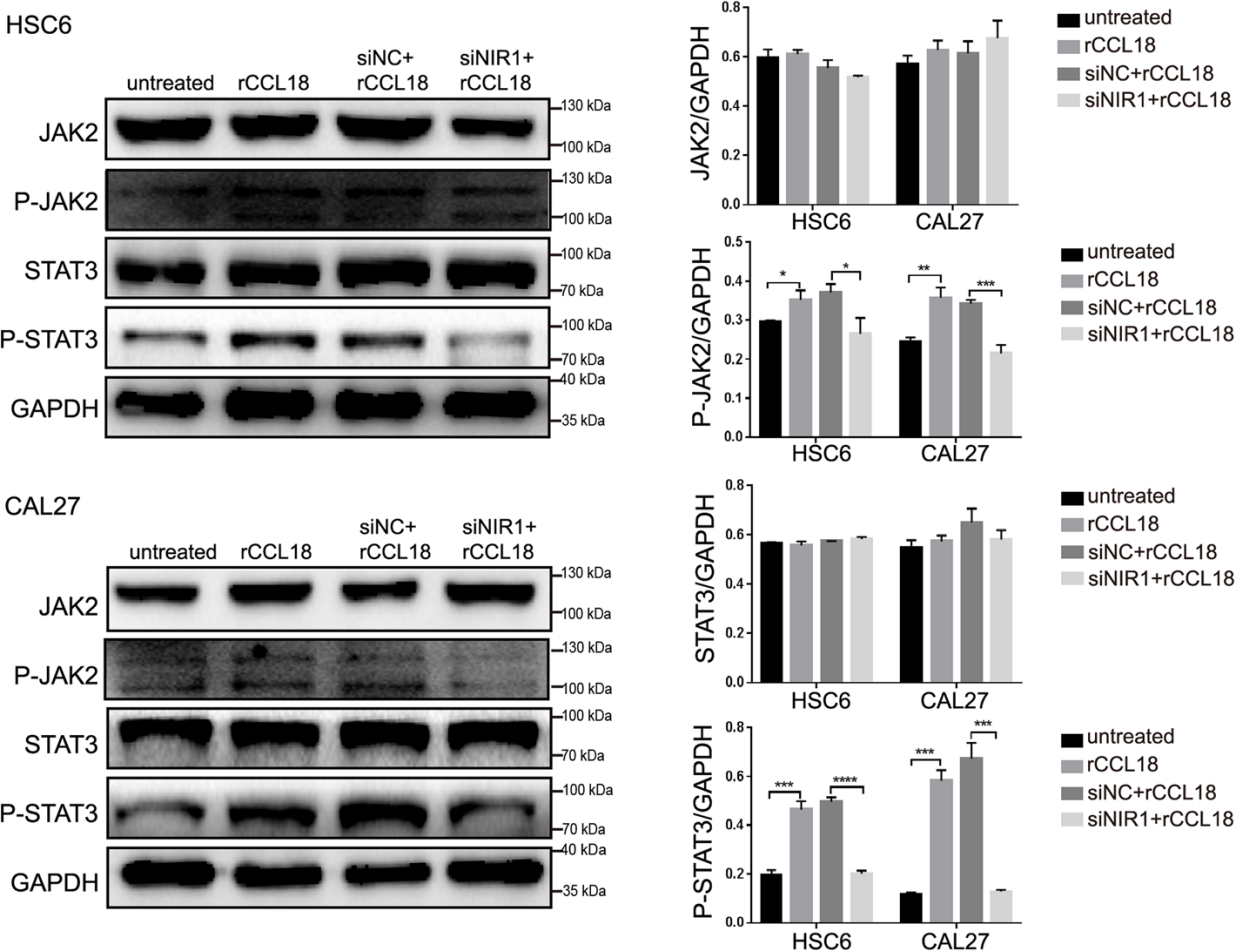
CCL18-NIR1 axis activates the JAK2/STAT3 pathway in OSCC cells. Western blotting showed that the expression of phosphorylated JAK2/STAT3 was detected in the rCCL18 group and siNC+rCCL18 group. siNIR1 abated the expression of phosphorylated JAK2/STAT3 of cells under the rCCL18 stimulated. GAPDH was used as the internal control. All data are presented as the mean ± SEM of the triplicate experiment. (*P < 0.05, **P < 0.01, ***P < 0.001, ****P < 0.0001 vs. control) . The full-length blots are presented in Supplementary Fig. S5

**Fig. 7:**
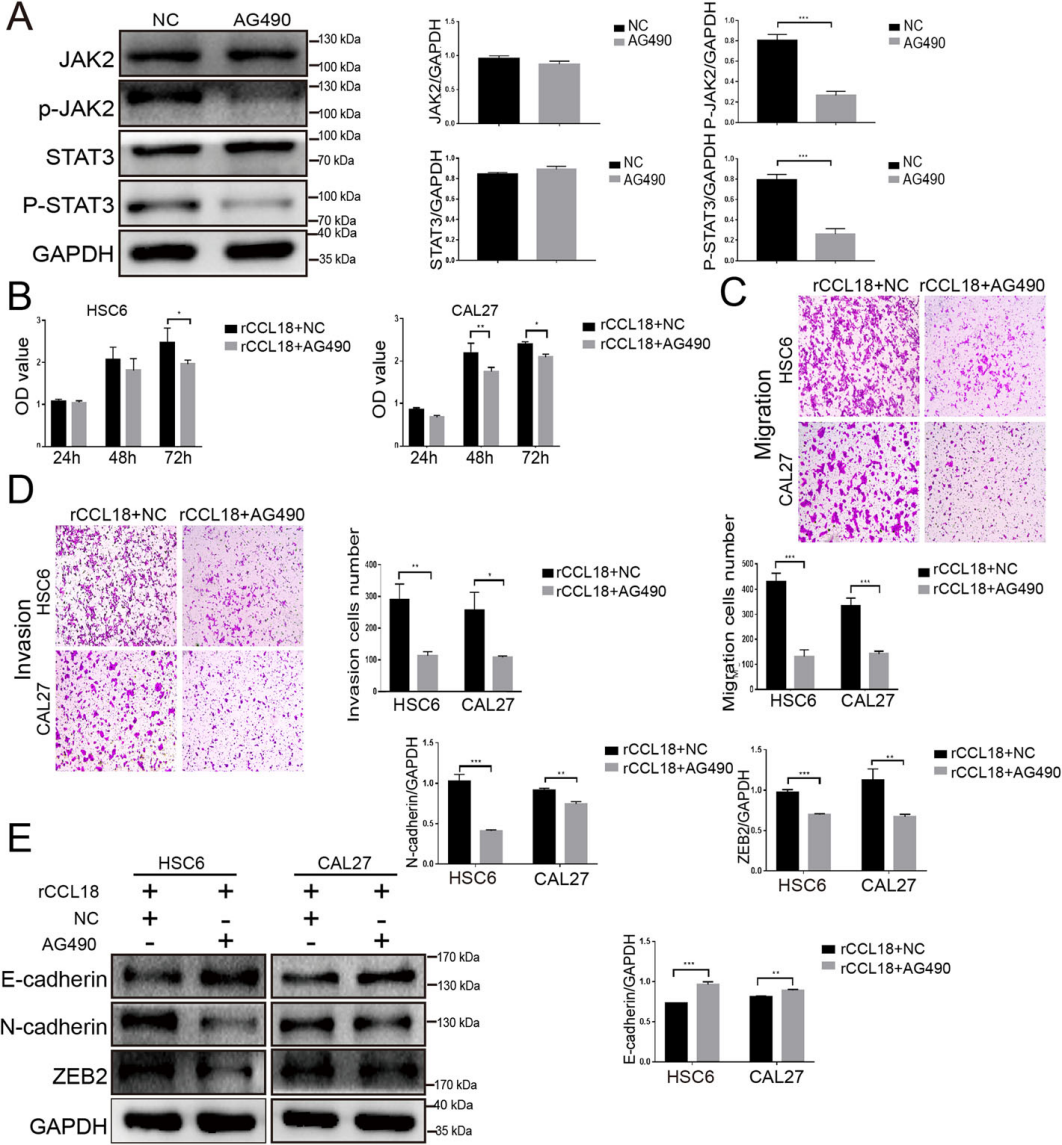
AG490 inhibited the CCL18-induced OSCC proliferation, migration, invasion, and EMT. **a** Western blotting showed that AG490 could effectively inhibit the expression of phosphorylation of both JAK2 and STAT3 in HSC6 cells. **b, c and d** Results of CCK8 and transwell demonstrated AG490 impaired the proliferation, migration, and invasion of rCCL18-stimulated OSCC cells. **e** The increased expression of E-cadherin and the decreased expression of N-cadherin and ZEB2 were found in the rCCL18 + AG490 group when compared with the rCCL18 + NC control group. (*P < 0.05, **P < 0.01, ***P < 0.001, vs. control). The full-length blots are presented in Supplementary Fig. S6

The JAK2/STAT3 pathway is known to be involved in the growth and EMT process in OSCC. As shown in Fig. 6 and Fig. S5, P-JAK2 (Tyr1007/1008) and P-STAT3 (Tyr705) levels were found increased, but there was no change in JAK2 and STAT3 protein expression levels in rCCL18-treated OSCC cells. Silencing NIR1 in HSC6 and CAL27 cells could reverse the activating effect of rCCL18 on JAK2 and STAT3. To further confirm the role of JAK2/STAT3 signaling pathway in CCL18-induced effects on OSCC, AG490, the JAK2-specific inhibitor, was used. 20 μM AG490 could significantly attenuate the phosphorylation of JAK2 and STAT3 in HSC6 cells but had no markedly effect on the total protein expression (Fig. 7a, Fig. S6). In addition, functional assessments showed that the proliferation, migration and invasion of OSCC cells (HSC6 and CAL27) in AG490 + rCCL18 group were obviously decreased compared with the rCCL18 group (Fig. 7b-d). Moreover, AG490 also reversed the decreased expression of E-cadherin, increased expression of N-cadherin and ZEB2 in OSCC cells, which were caused by rCCL18 (Fig. 7e, Fig. S6). Thus, these results indicated that both AG490 and siRNA-NIR1 abrogated the effect of CCL18 on OSCC cells. All these findings taken together indicated that the CCL18-NIR1 axis could promote the malignant progression of OSCC by activating the JAK/STAT3 signaling pathway.

## Discussion

Our previous work showed that CCL18, that was predominantly secreted by oral cancer cells, could promote the malignant progression of OSCC [6]. The data in this study delineated the mechanism of CCL18-induced OSCC cell proliferation, migration, and invasion, including the overexpressed functional receptor NIR1 and the activation of JAK2/STAT3 signaling pathway.

Previous research has shown aberrant expression patterns of NIR1 in numerous types of tumors including breast cancer, non-small cell lung cancer (NSCLC), and hepatocellular carcinoma (HCC) [18–20]. In 2011, NIR1 was validated to be a functional receptor of CCL18, and its dysregulation was noted to be involved in CCL18-induced calcium influx and chemotaxis in breast cancer [18]. Another study investigating NSCLC showed a significant correlation between NIR1 and CCL18, and also validated the role of CCL18-NIR1 in enhancing the malignancy of NSCLC cells [19]. Apart from being noted as aberrantly expressed in cancers, NIR1 overexpression has been associated with the clinical stage and histological grade of cancer in HCC [20]. In the present study, NIR1 was found as significantly upregulated in OSCC tissues and cell lines (i.e., HSC6, Cal27 and SCC9) compared with control adjacent normal tissues and HOK cells. In addition, clinical analysis of 43 OSCC patients showed that the expression of NIR1 was higher in TNM stage III/IV samples compared with TNM stage I/II samples, and, in addition, the NIR1 expression was significantly correlated with the expression levels of CCL18. These findings together indicated that the overexpression of NIR1 might be an indicator of the malignant progression of oral cancer.

Apart from NIR1, CCR8 and CCR6 have also been validated as CCL18 receptors in existing cancer research. In 2013, CCR8 was first identified to be the binding receptors of CCL18 in a study by Islam et al [21]. Subsequent research showed the involvement of the CCL18-CCR8 in enhancing the migration, invasion, and EMT of bladder cancer [12]. In addition to CCR8, CCR6 was found to be overexpressed in many pancreatic ductal cancer cells lines (e.g., PANC-1、CAPAN-2 and SW1990) [22]. However, the present study didn’t find significant differences in the expression levels of CCR8/CCR6 between OSCC tissues/cell lines and corresponding control samples. Considering such a finding, NIR1 was selected as the research focus of the present study and investigated further.

The CCL18-NIR1 axis has been shown to activate the intracellular calcium signaling and further promote the proliferation, metastasis and EMT in lung, liver, and breast cancer cells [19, 20, 23]. In accordance with these results, the present study showed si-NIR1 impaired CCL18-induced proliferation, migration, and invasion of HSC6 and CAL27 cells. Also in agreement with previous research, evidence of potential involvement of NIR1 in CCL18-induced metastasis in OSCC was shown by the reversing effect of NIR1 silencing on the rCCL18-induced expression patterns of EMT-related markers (e.g., E-cadherin, N-cadherin, and ZEB2). EMT is a key step involved in the progression of primary tumors toward metastasis. The upregulation of N-cadherin which is followed by the downregulation of E-cadherin is considered a hallmark of EMT. E-cadherin, as the main marker of epithelial cells, is critical for maintaining cell junction and cytoskeleton stability [24]. The replacement of E-cadherin by N-cadherin indicates an alteration of protein components of the cell connections and the enhancement of cell mobility [25]. The transcription factor ZEB2 (Zine Finger E-Box Binding Homebox 2) has been shown to be a master regulator and significant intermediate hub in the EMT signaling network. It has a crucial role in inducing EMT during the tumor progression and can recruit specific chromatin-modifying and -remodeling complexes to the promoter of specific genes like E-cadherin to silence their expression [26].

The JAK2/STAT3 signaling pathway is an evolutionarily conserved signaling pathway and is implicated in the tumor growth and metastasis of OSCC [14, 27]. The activation of JAK2 protein kinase can catalyze STAT3 protein phosphorylation which plays a role in regulating the expression of oncogenic genes [28]. E-cadherin, N-cadherin and ZEB2 are generally regarded as the downstream molecules of the JAK2/STAT3 signaling pathway [29–31]. Several studies have shown that JAK2/STAT3 can be activated by several chemokines including CXCL3, CCL20, CXCL9, and IL-6 [32–35]. However, the interplay between CCL18 and the JAK2/STAT3 signaling pathway remains unknown. Here, we found that the expression of P-JAK2 and P-STAT3 was increased in rCCL18-stimulated oral cancer cells. Whereas, the stimulatory effect of CCL18 on JAK2/STAT3 activation in OSCC was markedly diminished by siNIR1 treatment. Tyrphostin AG490 as the JAK specific inhibitor, restrains phosphorylation of JAK2 and STAT3, and subsequently decreases the expression of downstream targets, as well as mitigates the biological effects mediated by JAK2/STAT3 signaling pathway [36, 37]. A study investigating bladder cancer showed that AG490 could inhibit cell growth and invasion, as well as induce cell apotosis and cycle arrest by inhibiting the activation of the JAK2/STAT3 signaling pathway [38]. The present study determined that AG490 can abrogate the effects of CCL18 on OSCC cells, which is consistent with the effects of si-NIR1. All these results suggested that JAK2/STAT3 signaling contributes to the CCL18-NIR1 mediated proliferation and migration in OSCC.

It is worthwhile to clearly state the chief limitation of this study. The 5-year survival rate of OSCC patients was not analyzed in this study since several patients failed to participate in the follow-up appointments. However, this study also provides implications for future research. First, the molecular mechanisms identified in this study could be used as therapeutic targets for design of gene delivery therapeutics in OSCC. In addition, the identification of these critical mechanisms will also advance the development of multi-target drugs; however, such drugs could synergistically work conventional chemotherapeutic agents remains a question. Furthermore, molecular markers of metastasis could be regarded as novel evaluation indices for the prognosis of OSCC and testing kits based on these may be developed for early diagnosis and risk profiling.

## Conclusion

To conclude, NIR1 was identified to be upregulated in OSCC and associated with an advanced tumour stage. The CCL18-NIR1 axis was found to regulate the proliferation, metastasis, and EMT of OSCC cells by activating the JAK2/STAT3 signalling pathway. The signaling axis might be a novel therapeutic target for counteracting progression in oral cancer.

## Supplementary information

**Additional file 1: Figure S1.** The high expression of NIR1 in OSCC. Uncropped full-length blot images for Fig. 1 (B,D). The cropped blots were marked with red frame.

**Additional file 2: Figure S2.** The expression of CCR8 and CCR6 in OSCC. Uncropped full-length blot images for Fig. 2 (B, C). All these samples derived from the same experiment and blots were processed in parallel. The cropped blots were marked with red frame.

**Additional file 3: Figure S3.** The expression of NIR1 in HSC6 cells which were transfected with different siRNA segments. Uncropped full-length blot images for Fig. 3 (B). The cropped blots were marked with red frame

**Additional file 4: Figure S4.** CCL18-NIR1 axis promoted the EMT of OSCC cells. Uncropped full-length blot images for Fig. 5 (C). The expression of ZEB2, E-cadherin, and N-cadherin in the blots was marked with red frame. The proteins of the other lanes were not related to this study. All samples derived from the same experiment and blots were processed in parallel

**Additional file 5: Figure S5.** CCL18-NIR1 axis activated the JAK2/STAT3 signaling pathway. Uncropped full-length blot images for Fig. 6. The cropped blots were marked with red frame. The proteins of the other lanes were not related to this study. All samples derived from the same experiment and blots were processed in parallel.

**Additional file 6: Figure S6.** AG490 inhibited the CCL18 induced OSCC EMT. Uncropped full-length blot images for Fig. 7 (A,E). **A** The impact of AG490 on regulating the components of the JAK2/STAT3 signaling pathway. **E** The expression of EMT markers. The cropped blots were marked with red frame. The proteins of the other lanes were not related to this study. All samples derived from the same experiment and blots were processed in parallel.

## Data Availability

The datasets used and/or analysed during the current study are available from the corresponding author on reasonable request.

## Abbreviations

OSCC: Oral squamous cell carcinoma
CCL18: Chemokine (C-C motif) ligand 18
NIR1: N-terminal domain interacting receptor 1
CCR8: C-C chemokine receptor type 8
CCR6: C-C chemokine receptor type 6
rCCL18: Recombinant CCL18
JAK2/STAT3: Janus kinase 2/signal transducers and activators of transcription 3
P-JAK2: Phosphorylation of JAK2
P-STAT3: Phosphorylation of STAT3
EMT: Epithelial-mesenchymal transition
