# Supplementary figures and images for: CCL18-NIR1 promotes oral cancer cell growth and metastasis by activating the JAK2/STAT3 signaling pathway
Source: BMC Cancer. 2020 Jul 8;20:632. doi: 10.1186/s12885-020-07073-z (PMC7346480; doi:10.1186/s12885-020-07073-z)

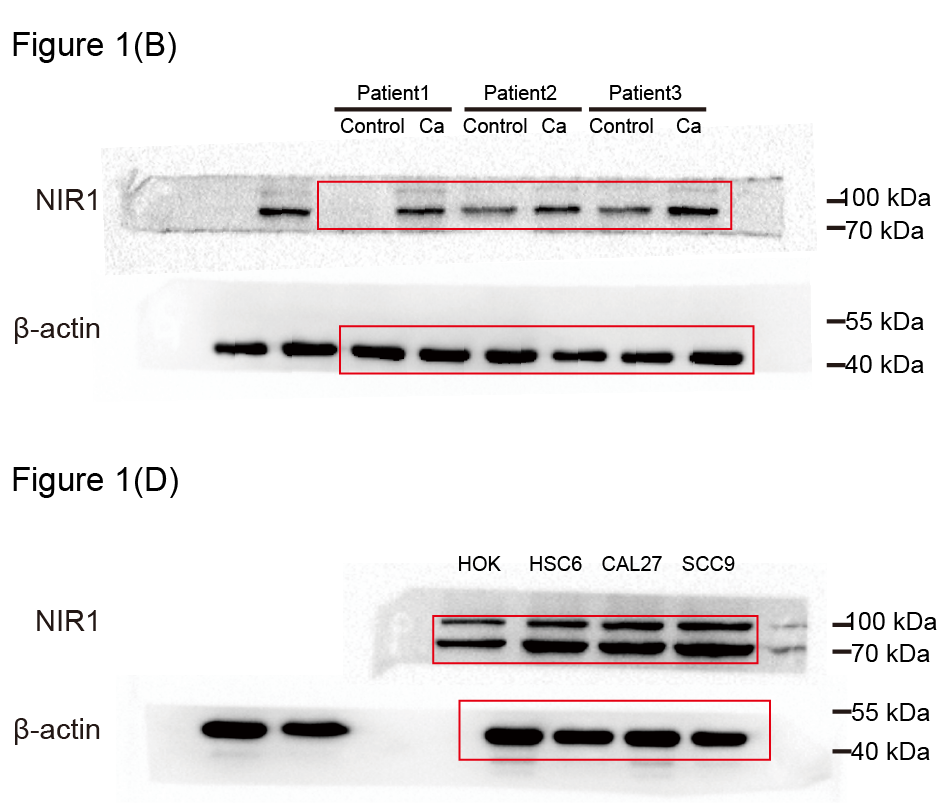

Supplement: Supplementary file 1 — Additional file 1: Figure S1. The high expression of NIR1 in OSCC. Uncropped full-length blot images for Fig. 1 (B,D). The cropped blots were marked with red frame. [file 12885_2020_7073_MOESM1_ESM.tif]

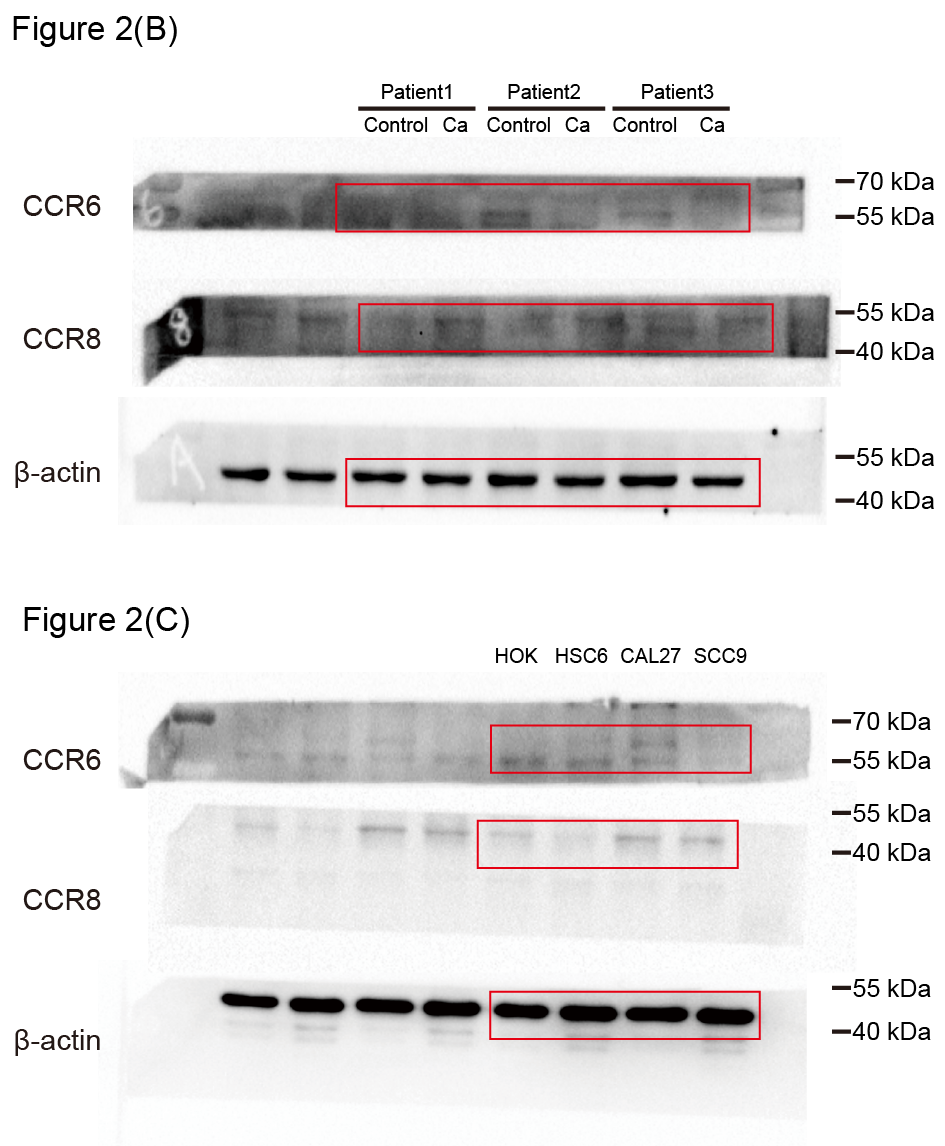

Supplement: Supplementary file 2 — Additional file 2: Figure S2. The expression of CCR8 and CCR6 in OSCC. Uncropped full-length blot images for Fig. 2 (B, C). All these samples derived from the same experiment and blots were processed in parallel. The cropped blots were marked with red frame. [file 12885_2020_7073_MOESM2_ESM.tif]

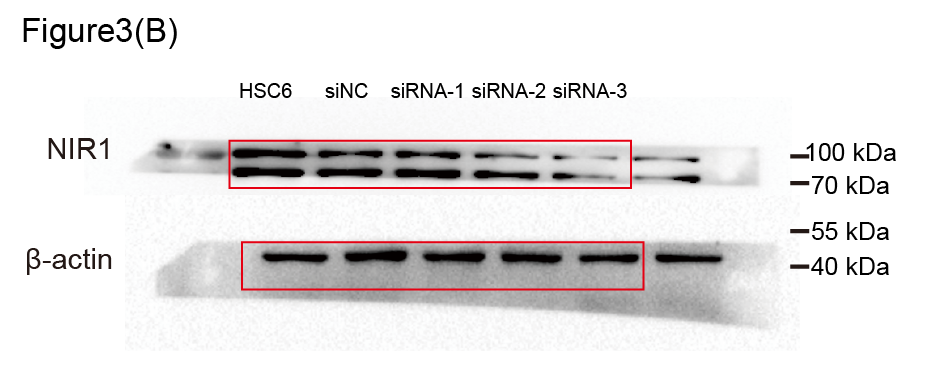

Supplement: Supplementary file 3 — Additional file 3: Figure S3. The expression of NIR1 in HSC6 cells which were transfected with different siRNA segments. Uncropped full-length blot images for Fig. 3 (B). The cropped blots were marked with red frame [file 12885_2020_7073_MOESM3_ESM.tif]

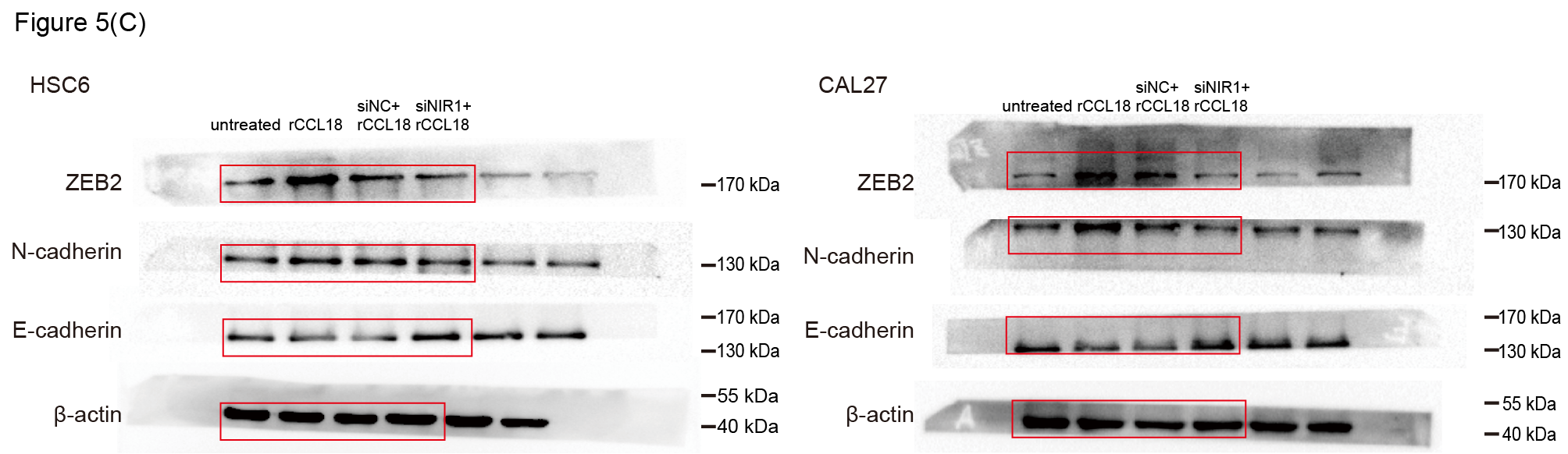

Supplement: Supplementary file 4 — Additional file 4: Figure S4. CCL18-NIR1 axis promoted the EMT of OSCC cells. Uncropped full-length blot images for Fig. 5 (C). The expression of ZEB2, E-cadherin, and N-cadherin in the blots was marked with red frame. The proteins of the other lanes were not related to this study. All samples derived from the same experiment and blots were processed in parallel [file 12885_2020_7073_MOESM4_ESM.tif]

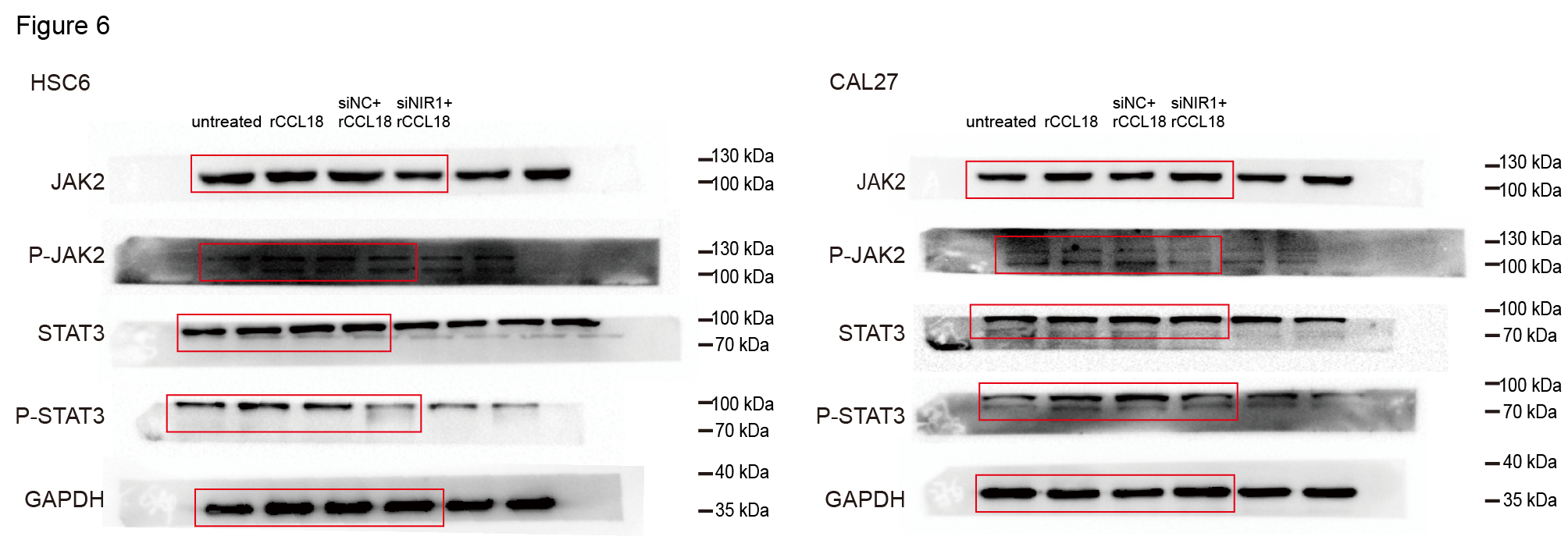

Supplement: Supplementary file 5 — Additional file 5: Figure S5. CCL18-NIR1 axis activated the JAK2/STAT3 signaling pathway. Uncropped full-length blot images for Fig. 6. The cropped blots were marked with red frame. The proteins of the other lanes were not related to this study. All samples derived from the same experiment and blots were processed in parallel. [file 12885_2020_7073_MOESM5_ESM.tif]

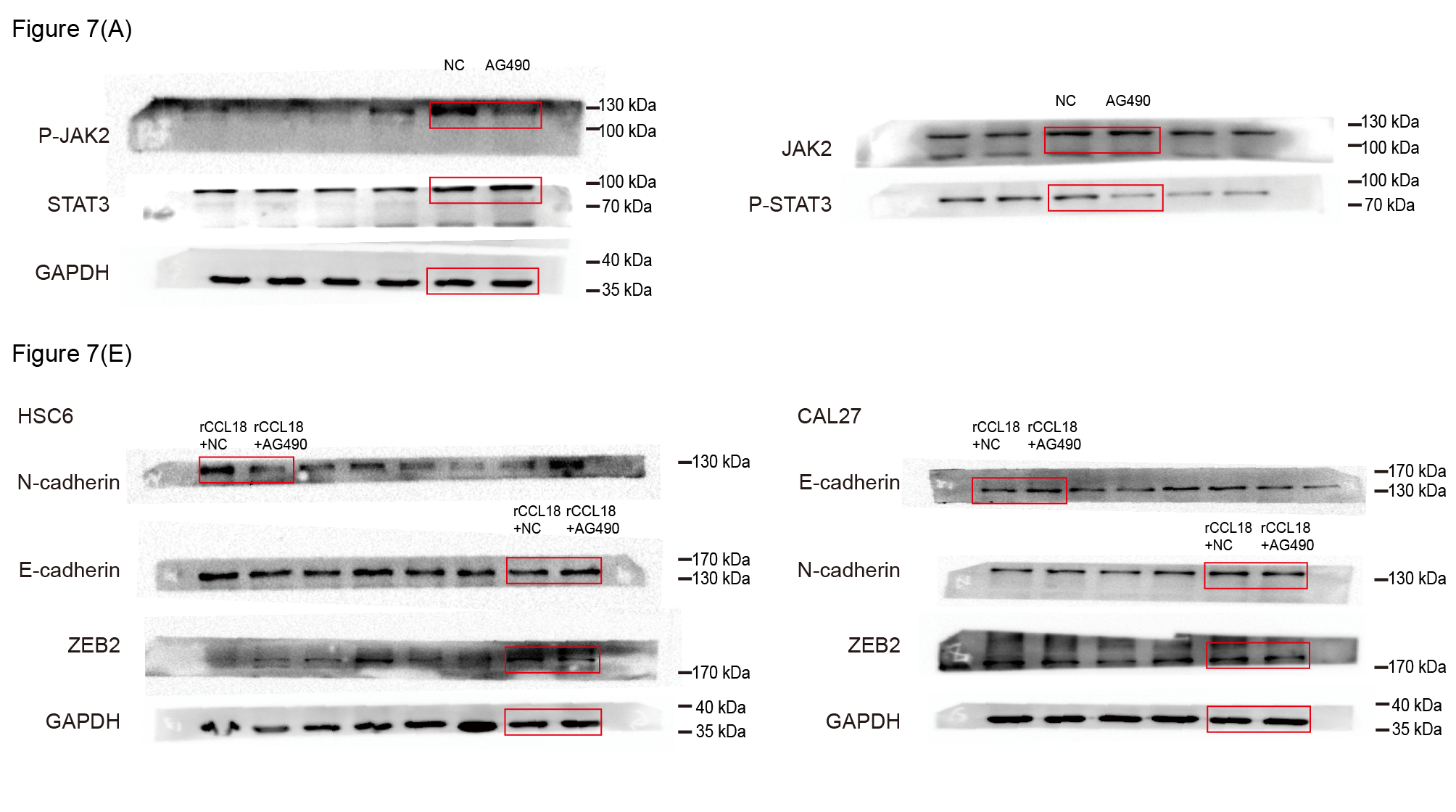

Supplement: Supplementary file 6 — Additional file 6: Figure S6. AG490 inhibited the CCL18 induced OSCC EMT. Uncropped full-length blot images for Fig. 7 (A,E). A The impact of AG490 on regulating the components of the JAK2/STAT3 signaling pathway. E The expression of EMT markers. The cropped blots were marked with red frame. The proteins of the other lanes were not related to this study. All samples derived from the same experiment and blots were processed in parallel. [file 12885_2020_7073_MOESM6_ESM.tif]
